# Cost Consequence Analysis of Non‐Surgical Treatment Approaches for Patients With Periodontitis

**DOI:** 10.1111/jcpe.70154

**Published:** 2026-06-19

**Authors:** Anna Liss, Max Petzold, Cristiano Tomasi, Kajsa H. Abrahamsson, Maria Welander

**Affiliations:** ^1^ Department of Periodontology, Institute of Odontology, the Sahlgrenska Academy University of Gothenburg Gothenburg Sweden; ^2^ Clinic of Periodontology, The Public Dental Service, Region Västra Götaland Gothenburg Sweden; ^3^ School of Public Health and Community Medicine, Institute of Medicine, the Sahlgrenska Academy University of Gothenburg Gothenburg Sweden

**Keywords:** cost consequence analysis, dental hygienist, effectiveness study, general dentistry, non‐surgical periodontal therapy

## Abstract

**Aim:**

To analyse and compare cost consequences of two evidence‐based non‐surgical periodontal treatment protocols performed by dental hygienists (DHs) in general practice.

**Materials and Methods:**

Six‐hundred and fifteen patients were included. Treatment approaches compared were (i) conventional non‐surgical therapy (CNST), often performed at multiple sessions, and (ii) a ‘full‐mouth’ concept called guided periodontal infection control (GPIC), with initial focus on patient education followed by one session of ultrasonic instrumentation. A cost consequence analysis was conducted regarding the two approaches, including treatment costs, travelling costs and productivity costs, as well as non‐monetary consequences (closed pockets and bleeding on probing). In data analysis, the 95% confidence intervals were obtained using the percentile method based on 5000 bootstrap replications.

**Results:**

Treatment costs were higher for CNST compared to GPIC by €60 (€359.5 [95% CI: 345.5–374.2] vs. €299.4 [95% CI: 289.3–309.1]). Productivity costs related to treatment were also greater in CNST by +€15. Travel costs showed minor differences (+€1 for CNST). Non‐monetary outcomes showed no significant differences between groups.

**Conclusion:**

CNST was more costly than GPIC while maintaining comparable clinical outcomes at 6 months. When designing policy and practice for non‐surgical treatment approaches, both evidence‐based practice and how shared resources are best used should be acknowledged.

## 1 Introduction

Periodontitis represents one of the most prevalent chronic, non‐communicable inflammatory diseases affecting humans, with a prevalence of severe disease of about 11% in adult populations. Hence, periodontitis constitutes a major public health problem, for which cost‐effective prevention and treatment approaches are of utmost importance (Bishop 2021; Dumitrescu 2016; N. Kassebaum et al. 2014; Sanz et al. 2020). Prevention and treatment of periodontitis aim to establish infection control, which is achieved through (i) effective self‐care by the patient and (ii) mechanical instrumentation of affected tooth and root surfaces (Sanz et al. 2020).

The guidelines by the European Periodontal Federation (EFP) as well as Swedish national guidelines emphasise the importance of patient education for adequate patient self‐care and recognise two different approaches for the initial treatment phase (Steps 1–3) of mechanical non‐surgical instrumentation in the treatment of patients with periodontitis (Sanz et al. 2020; Socialstyrelsen 2011, 2022). The approaches are (i) conventional instrumentation, often performed section‐wise at multiple sessions, using hand instruments and/or ultrasonic instruments, and (ii) a ‘full‐mouth’ concept, where instrumentation is performed in one or two single sessions using ultrasonic instruments (Sanz et al. 2020; Socialstyrelsen 2011, 2022). Both approaches have been shown as clinically effective in numerous randomised controlled trials (RCTs), that is, efficacy studies, most often performed in specialist settings under optimal conditions (Eberhard et al. 2015; Suvan et al. 2020). Still, it has been argued that the ‘full‐mouth’ approach may have health‐economic benefits in terms of (less) invasiveness and time consumption (Koshy et al. 2005; Wennström et al. 2005). Furthermore, the importance of evaluating methods when treatments are performed in everyday practice, that is, effectiveness studies, has been emphasised (SBU 2004; Socialstyrelsen 2011).

To meet the requests as stated above, a randomised clinical field study was initiated by our group to evaluate the effectiveness of the two evidence‐based approaches in the non‐surgical treatment of patients with periodontitis. The treatment outcomes from this field study (Liss et al. 2021; Tomasi et al. 2022) were in line with previous RCT studies (Eberhard et al. 2015; Suvan et al. 2020). However, the ‘full‐mouth’ concept proved to be more time‐efficient, as it took significantly less time to achieve healthy conditions (Tomasi et al. 2022). Still, a more careful cost consequence analysis can provide valuable information in relation to resource utilisation. Health economic evaluations are of importance to select the most effective treatment method while utilising the least possible resources (M. F. Drummond 2015; Husereau et al. 2022). Therefore, the specific aim of this study was to analyse and compare the cost consequences of two evidence‐based non‐surgical periodontal treatment protocols, performed by dental hygienists (DHs) in general practice.

## 2 Materials and Methods

The data in the present cost consequence analysis originate from a randomised clinical field study within the Public Dental Service in region Västra Götaland (VG), Sweden (Tomasi et al. 2022). The study protocol was approved by the Regional Ethical Review Board of Gothenburg, Sweden (Dnr: 288‐13). All participants signed an informed consent form before entering the study, and all study procedures were performed in accordance with ethical rules and principles (Holm 2013). The study was registered at ClinicalTrials.gov (NCT02168621). The CHEERS guidelines were followed for reporting of health economic analyses (Husereau et al. 2022).

### 2.1 Interventions—Treatment Protocols

The two non‐surgical treatment protocols evaluated were (i) conventional non‐surgical therapy (CNST) and (ii) guided periodontal infection control (GPIC), as described in Table 1.

**TABLE 1:** Description of the two non‐surgical periodontal treatment protocols evaluated in the clinical field study (Tomasi et al. 2022).

Conventional non‐surgical therapy (CNST)	Guided periodontal infection Control (GPIC)
CNST, that is, conventional non‐surgical treatment, typically consists of several (3–4) sessions and with patient education and subgingival instrumentation integrated. No specific instructions were given to the DHs regarding time or choice of instruments.	GPIC, comparable to a ‘Full‐mouth’ concept, is a stepwise protocol designed to guide both the patient and the therapist through the treatment process. GPIC have an initial focus on patient education to establish adequate oral hygiene. At a plaque score ≤ 30%, subgingival instrumentation was performed in a single session (~1 h) using ultrasonic instruments.
**Follow up and re‐treatment for both protocols**	
At the 3‐month follow‐up residual pockets (PPD ≥ 5 mm and presence of BOP) were identified and re‐instrumented, without restrictions regarding time or choice of instruments. Clinical outcomes were evaluated at 6 months.	

Abbreviations: BOP, bleeding on probing; PPD, probing pocket depth.

### 2.2 Study Population

The patients enrolled in the study (*n* = 615), all diagnosed with periodontitis, were treated by 95 registered DHs, at 59 general dental clinics in the Public Dental Service, VG (for details see Tomasi et al. 2022). The demographic and clinical characteristics of the patients are presented in Table 2. Baseline differences were observed between the two treatment groups with respect to the three levels of disease severity (stage II [probing pocket depth, PPD ≤ 5 mm]; stages III and IV [≤ 3 teeth with PPD ≥ 6 mm]; and stage III and IV [≥ 4 teeth with PPD ≥ 6 mm]), Table 2.

**TABLE 2:** Characteristics of the study sample at baseline. Presented as mean, range, 95% CI and % (numbers). Total number of participants (*n*) = 615.

Demographical and clinical variables	CNST (*n* = 335)	GPIC (*n* = 280)
Age, mean (range)	53.3	53.3
Gender, %		
Female	43.6	45.7
Male	56.4	54.3
Occupation, %		
White‐collar worker	35.5	34.3
Blue‐collar worker	35.8	38.6
Retired	21.5	20.4
Student	0.9	1.4
Other	6.3	5.4
Teeth status at baseline, mean (95% CI)		
No. of teeth	26.5 (26.2–26.7)	26.0 (25.7–26.3)
No. of teeth with PPD ≥ 5 mm	11.5 (11.0–12.1)	10.4 (9.9–10.9)
No. of teeth with PPD ≥ 6 mm	5.2 (4.7–5.6)	3.9 (3.5–4.3)
Periodontal disease severity at baseline, %		
Stage II (PPD ≤ 5 mm)	10.4	18.2
Stage III‐IV (≤ 3 teeth with PPD ≥ 6 mm)	31.0	36.1
Stage III‐IV (≥ 4 teeth with PPD ≥ 6 mm)	58.6	45.7

Abbreviations: CNST, conventional non‐surgical therapy; GPIC, guided periodontal infection control.

### 2.3 Study Perspective and Outcomes

The current cost consequence analysis (CCA) reports the primary outcome from a health care perspective: *treatment costs* related to the non‐surgical periodontal treatment (costs associated with dental materials and personnel, inclusive of overhead costs). As a complementary analysis, the CCA also reports costs from a societal perspective: patients' *costs for travelling* to visits to the dental offices and *productivity costs* due to travelling time and time for visits to the dental offices. Productivity costs were estimated using a human capital approach, consistent with the adopted societal perspective (M. F. Drummond 2015). Only individuals active in the labour market were included in the productivity cost calculations. Unemployed individuals, retirees and students were not assigned.

In addition, clinical categories included in the CCA were closed pocket (i.e., PPD ≤ 4 mm) and bleeding on probing (BOP), as earlier presented by Tomasi et al. (2022). Use of CCA (costs and clinical outcomes separately, rather than combined into an overall measure) allows decision makers to assess the relative importance of each outcome.

(Drummond et al. 2006).

### 2.4 Data Collection

Data collection was performed in three steps: identifying the cost items, quantifying the resource use and valuing the resource use to arrive at costs per resource consumed and total costs. The report analyses treatment costs, travelling costs and productivity costs (productivity costs were only calculated for the participants categorised as employed) of the two non‐surgical treatment concepts.

Treatment time was quantified by notes taken by the DHs at each treatment session, that is, patient education (information, instruction and motivation) and mechanical instrumentation of the affected teeth and root surfaces as well as oral hygiene check‐up. Treatment time was on average 161 and 134 min in CNST and GPIC, respectively (Table S1). In addition, questionnaire data regarding travel distance (Table S2), travelling time (Table S1) and transport mode (Tabel S2) to the dental office were quantified by notes taken by the DHs at baseline. The costs for different modes of transport were retrieved from public databases such as the Swedish Automobile Association (Table S2). Travel distance was on average 68 km in CNST and 66 km in GPIC. The total travelling time was on average 114 min.

The patients' estimated average hourly wage (including wage taxes, assumed to be 40%) was based on the average monthly wage in Sweden divided by 165 h (SCB 2026). To calculate the value of non‐employees' leisure time included in a sensitivity analysis, the average income tax rate (assumed a standard 40%) was deducted from the average hourly wage. The average hourly wage for employees was €33, and the average value of non‐employed participants was assumed at €14.

Data regarding the hourly rate of treatment by a DH (the rate reflects the full cost of providing 1 h of clinical work, including staff‐related expenses, clinic infrastructure and organisational overhead^1^) were retrieved from the national Public Dental Service (‘The Swedish Public Dental Service’ 2025). The hourly rate was on average €134 (Table S3).

### 2.5 Description of Cost Calculation

Cost items (price, quantity and total values) included in the analysis are presented in Table 3.

**TABLE 3:** Price and quantity table with cost items, quantities and values regarding Treatment cost, Travelling cost and Productivity cost of non‐surgical periodontal therapy (steps 1~3, Sanz et al. 2020) (*n* = 615).

	Price (*P*) mean (range)		Treatment CNST			Treatment GPIC		
			Quantity (*Q*)	Total mean (95% CI)		Quantity (*Q*)	Total value mean (95% CI)	
Cost item	(€)	(SEK)		(€)	(SEK)		(€)	(SEK)
Treatment cost	134.1	1453.6	2.7 h	359.5 (345.5–374.2)	3897.3 (3744.9–4056.6)	2.2 h	299.4 (289.3–309.1)	3245.7 (3136.5–3350.6)
Travelling cost	0.4	3.9	68.0 km	24.5 (23.7–25.3)	265.2 (256.3–273.9)	65.5 km	23.6 (22.9–24.2)	255.5 (247.9–262.8)
Productivity cost^a^	32.6	353.0	4.6 h	150.4 (146.5–154.6)	1630.7 (1588.3–1676.1)	4.2 h	135.7 (133.0–138.5)	1471.1 (1441.3–1501.0)
Total	—	—	—	538.9	5793.2	—	460.7	4972.3

*Note:* € 1 = SEK 10.84 (in March 2025).

Abbreviations: €, Euro; CNST, conventional non‐surgical therapy; GPIC, guided periodontal infection control; SEK, Swedish crowns.

^a^ Individuals in paid employment.

Treatment cost was calculated by multiplying the unit cost (*P*), that is, the DH′s hourly rate, with the quantity (*Q*), that is, the total treatment time in hours.

DHhourly rate×treatment time

Travelling cost was calculated by multiplying (*P*), that is, the standard average cost of transport, with (*Q*), that is, the total travelling distance. The average travel distance was calculated as an average for all participants, an average that was multiplied by the number of visits per person to GPIC and CNST, respectively.

Cost of transport×travelling distance

Productivity cost was calculated by multiplying (*P*), that is, the hourly wage (for employees), with (*Q*), that is, the total treatment and travelling time (for employees). The total treatment and travel time is calculated by using the average travel time for the two groups plus the total treatment time for CNST and GPIC, respectively.

$$\text{Time}\;\mathrm{off}\;\text{work}\times \text{gross wage}\;\left(i.e.,\text{employer costs}\right)$$

In this cost analysis, we assume that all personnel involved have the professional training and expertise required to perform the procedures; therefore, training costs are not included in the calculations. To reflect real‐world conditions, we combine the two groups' travel distance, transportation cost and wages, as these elements were not related to treatment group randomisation and do not affect the clinical outcomes of the treatment options.

The focus of the cost analysis was to illustrate the cost implications of the differences in visits (and treatment time) between the two treatment options. Our complementary societal perspective analysis therefore includes travel costs based on the number of visits, as well as productivity costs associated with treatment time and travel time. We intend to evaluate the practical impact at the system level of implementing these methods in routine clinical practice, rather than estimating costs for individual patients.

### 2.6 Currency, Price Date and Conversion Date

The clinical field study (Tomasi et al. 2022) was conducted during 2014–2017. However, the current cost analysis is based on the price level of the year 2025. The currency for all calculations is given in € (Euros) and SEK (Swedish Crowns), €1 = SEK10.84 (in March 2025). SEK values are presented only in the tables, not in the text. No discounting of costs was performed because of the short 1‐years time horizon.

### 2.7 Characterising Heterogeneity, Distributional Effects and Uncertainty

Two deterministic one‐way sensitivity analyses were performed to reflect uncertainty in the estimates: *Treatment cost* based on disease severity at baseline (Table S4), and including the value of leisure time for ‘retired, students and others’ in the *Productivity cost* (Table S5). Disease severity at baseline (Table 2) was considered because there was a significant difference between GPIC and CNST at the start of the clinical study (Tomasi et al. 2022), which may have an impact on the treatment intensity. Since productivity costs depend on labour market participation, we tested the robustness of the productivity cost results based on alternative assumptions for patients in paid employment compared to retired patients.

### 2.8 Data Handling and Analysis

Patients included in the present study had comparable background characteristics (employment, travel distance, travel time and mode of transportation). Included study variables were analysed using mean values, and for comparison between groups, bootstrap confidence intervals were constructed by resampling the original data with replacement (5000 replications). The 95% confidence intervals correspond to the 2.5th and 97.5th percentiles of the empirical bootstrap distribution of the mean difference. Because cost data are often skewed, bootstrap methods provide a way to estimate uncertainty without relying on assumptions of normality (M. F. Drummond 2015).

SPSS (The Statistical Product Service Solution, version 28) was used for data management of descriptive and inferential statistics.

## 3 Results

The CCA included data based on 615 patients.

### 3.1 Treatment Costs

Treatment costs related to non‐surgical periodontal therapy were higher for CNST compared to GPIC, with additional costs of +€60 (€359.5 [95% CI: 345.5–374.2] compared to €299.4 [95% CI: 289.3–309.1] for CNST and GPIC, respectively), with CIs estimated using non‐parametric bootstrapping (5000 replications) (Table 4). On average, the treatment time for patients in CNST was 161 min (range 52–490 min) and in GPIC 134 min (range 21–250 min) (Table S2).

**TABLE 4:** Cost consequence analysis (CCA). Estimated cost per patient and clinical outcome (mean, 95% CI).

Cost category	CNST		GPIC		Incremental (CNST–GPIC)	
	(€)	(SEK)	(€)	(SEK)	(€)	(SEK)
Treatment cost	359.5 (345.5–374.2)	3897.3 (3744.9–4056.6)	299.4 (289.3–309.1)	3245.7 (3136.5–3350.6)	+60.1 (42.8–77.4)	+651.6 (464.2–838.6)
Societal perspective						
Travelling cost	24.5 (23.7–25.3)	265.2 (256.3–273.9)	23.6 (22.9–24.2)	255.5 (247.9–262.8)	+0.9 (−0.2 to 1.9)	+9.7 (−1.72 to 21.1)
Productivity cost	150.4 (146.5–154.6)	1630.7 (1588.3–1676.1)	135.7 (133.0–138.5)	1471.1 (1441.3–1501.0)	+14.7 (9.8–19.7)	+159.6 (106.2–213.2)

Clinical consequences (at 6 months)	(%)	(%)	Incremental difference (%)
Closed pocket^a^	71.5 (68.7–74.3)	69.3 (66.0–72.6)	+2.2 (−2.1 to 6.5)
BOP	34.0 (32.8–35.2)	36.8 (35.4–38.2)	‐2.8 (−2.8 to −1.0)

*Note:* € 1 = SEK 10.84 (in Mars 2025).

Abbreviations: €, Euro; BOP, bleeding on probing; CNST, conventional non‐surgical therapy; GPIC, guided periodontal infection control; PPD, probing pocket dept.; SEK, Swedish crowns.

^a^ PPD ≤ 4 mm.

### 3.2 Travelling Costs

The total travel costs to dental clinics showed only a minor difference between the groups: CNST gave a marginally higher cost of +€1 than GPIC (€24.5 [95% CI: 23.7–25.3] and €23.6 [95% CI: 22.9–24.2] for CNST and GPIC, respectively) (Table 4). Patients in CNST and GPIC visited the dental offices on average 4.1 times (range 1–9) and 4.0 times (range 1–5), respectively (Table S1).

### 3.3 Productivity Costs

Productivity costs related to treatment were greater in the CNST group, amounting to an extra €15 (€150.4 [95% CI: 146.5–154.6] and €135.7 [95% CI: 133.0–138.5] for CNST and GPIC, respectively) (Table 4). On average, the mean total treatment time for patients in CNST was 163.0 min (52.0–438.0 min) and in GPIC 135.9 min (20.50–250.0 min) (Table S1). The total time for treatment and travelling was on average 277.2 min (166.2–604.2 min) for CNST and 250.04 min (134.7–364.2 min) for GPIC.

### 3.4 Non‐Monetary Consequences in Terms of Clinical Outcomes

The non‐monetary consequences included in the CCA (Table 4) were closed pocket (i.e., a PPD ≤ 4 mm) and BOP (previously reported in Tomasi et al. 2022). The analysis showed an incremental difference between CNST and GPIC of 2.2% (95% CI: –2.1 to 6.5) for pocket closure and 2.8% (95% CI: −2.8 to −1.0) for BOP. The differences were not statistically significant between the two treatment groups.

### 3.5 Sensitivity Analyses

The sensitivity analysis for treatment costs by subgroups based on disease severity at baseline (Table S4) showed that treatment costs increased with higher disease severity in both treatment groups. For patients with stage II disease, costs were lower than for those with more advanced disease. In the most severe subgroup (stages III and IV with ≥ 4 teeth with PPD ≥ 6 mm), both CNST and GPIC showed the highest mean costs. Across all severity levels, GPIC consistently had lower mean costs compared to CNST.

The subgroup analysis for *Productivity cost* (Table S5) showed that patients in ‘paid employment’ had mean productivity cost amounting to €150 for CNST and €136 for GPIC. Among retired individuals, students and others without paid employment, productivity costs valued as leisure time were substantially lower, reflecting the lower value of time applied in this subgroup. In this cohort, CNST had a mean productivity cost of €62 and GPIC €56. The total productivity costs remained slightly higher in the CNST group (€212 compared to GPIC €191).

## 4 Discussion

The present cost consequence analysis found that the treatment costs more for CNST compared to GPIC (+€60). Productivity costs also differed between CNST and GPIC, in favour of GPIC. Regarding the travelling cost to the dental offices, no statistically significant difference between groups was detected. Non‐monetary consequences (closed pocket and BOP) included in the cost consequence analysis showed no incremental differences between the two treatment groups in the non‐surgical periodontal treatment.

As stated before, periodontitis is a public health issue (Bishop 2021; N. Kassebaum et al. 2014; Sanz et al. 2020). Globally, the total cost for dental diseases in 2019 was estimated as $710 billion, wherein the direct treatment cost was $387 billion (Jevdjevic and Listl 2025). Previous studies have shown that periodontitis contributes to a significant cost of dental disease, as teeth lost due to periodontitis may require reconstructive care (Botelho et al. 2021; Pattamatta et al. 2025; Sanz et al. 2020). Pattamatta et al. (2025) estimated the direct treatment costs of periodontitis at $104 billion and the indirect costs at $82 billion, findings based on earlier studies assessing the global burden of dental disease (Jevdjevic and Listl 2025; N. J. Kassebaum et al. 2017; Passarelli et al. 2020).

The current results show that the mean dental costs for non‐surgical periodontal therapy were significantly lower when applying the concept of GPIC compared to CNST (20%) with no statistical difference in clinical outcomes. If we look at this from a broader perspective, approximately 811,000 adult individuals received periodontal care in Sweden between 2021 and 2023 (SKaPa 2024), which corresponds to approximately 280,000 individuals per year.

Given that one treatment may potentially entail higher costs than another, it is important from a societal perspective to not choose treatment concepts simply out of habit, but by making active choices based on the best evidence and the cost of treatment. Being up‐to‐date and informed about the effect of care and the different approaches available can benefit both the patient and the clinic (in terms of resource use) as well as society (Botelho et al. 2021; M. F. Drummond 2015; Righolt et al. 2018; Tonetti et al. 2017).

However, to our best knowledge, there is limited information on the prevalence of applying a full‐mouth concept (e.g., GPIC) in practice. A report from 2014 (not published) regarding DHs′ (*n* = 302) work and work conditions in region VG, Sweden showed that 87% of the DHs worked according to a conventional non‐surgical therapy, while 13% applied a full‐mouth concept. The choice of treatment approach was reported to be based on patient preferences, severity of periodontitis and the patient's general health. Additionally, the scheduling system had an impact on how treatment was planned and carried out.

This present cost analysis showed that patients in GPIC and CNST made roughly the same number of visits to the dental offices. However, the GPIC group required significantly less time for mechanical treatment and incurred lower treatment costs compared to the CNST group (Tables 4 and S1). Nevertheless, a limitation that should be considered in the current study is the significant difference between GPIC and CNST in terms of disease severity at baseline (Table 2). Because of this, the need for treatment intensity may have varied and contributed to the differences in costs. However, the sensitivity analysis (Table S4) showed that regardless of disease severity, GPIC consistently had lower average costs compared to CNST.

In addition, it is important to note that the GPIC approach had a strong focus on patients' oral self‐care, with dedicated visits focusing on patient education and motivation for effective, self‐performed infection control. This educational focus is well supported by a substantial body of evidence, as effective oral self‐care is essential for successful non‐surgical periodontal treatment (Axelsson, Nyström, and Lindhe, 2004; Sanz et al. 2020). A previous study conducted by our research group, examining patient‐reported experiences and outcomes of non‐surgical treatment, found that patients accepted GPIC and CNST to a similar extent. Notably, one factor in the study emerged as particularly important in achieving successful treatment outcomes: ‘patients feeling actively involved in their care and decision‐making process’ (Liss et al. 2021). Overall, the patient's experience of treatment, combined with health economic considerations, is central to the choice of non‐surgical periodontal care strategies.

One might wonder whether we need to change policy and practice regarding existing guidelines for non‐surgical periodontal treatment methods. The treatment methods evaluated in the current study yielded comparable results from a clinical perspective (Tomasi et al. 2022), results that are in line with several efficacy studies within the field (Koshy et al. 2005; Loggner Graff et al. 2008; Suvan et al. 2020; Wennström et al. 2005). However, this study showed that GPIC has the advantage of being less time‐consuming and therefore less costly for the patient, the dental care system and society. With this as a starting point, dental professionals should not only consider evidence‐based practice when proposing a treatment but also consider how our shared resources are best used.

A limitation to consider is that, based on our data, we cannot comment on the cost related to non‐surgical periodontal treatment in a longer perspective. However, the primary aim of this study was to examine the cost implications of the two treatment methods during the initial treatment phase. Another limitation of the analysis is that the bootstrapping procedure was performed in the two treatment groups separately without considering any clustering, for example, at the DH/clinics due to small cluster sizes. Ignoring such clustering may lead to underestimated standard errors and overly narrow CIs. However, given the small cluster sizes, cluster bootstrapping was not pursued. Furthermore, given that productivity and travel costs were calculated using pooled group‐level averages, uncertainty in these estimates is likely underestimated. Hence, these results should be interpreted cautiously, which are presented as secondary/supplementary analyses.

Additionally, it should be noted that randomisation to the two treatment protocols (CNST and GPIC) was performed at the DH level (Tomasi et al. 2022). Hence, we cannot fully exclude the possibility that their recruitment of patients may have been influenced—consciously or unconsciously—by personal preferences or expectations regarding the assigned treatment protocol. Such recruitment bias could potentially affect the outcomes of periodontal therapy. Nevertheless, the earlier study by Tomasi et al. (2022) reported no systematic differences between the DHs that would indicate structural bias. Patients included in this report had comparable background characteristics. The patients were diagnosed with periodontitis, and, from an ethical point of view, they would have been offered treatment in accordance with one of the two treatment protocols even without participating in the study.

In conclusion, CNST was more costly than GPIC while maintaining comparable clinical outcomes at 6 months. When designing policy and practice for non‐surgical treatment approaches, both evidence‐based practice and how shared resources are best used should be acknowledged.

## Author Contributions

A.L. contributed to data collection, data analysis, interpretation of data and drafting of the manuscript. M.P. was involved in data analysis, interpretation of data and drafting of the manuscript. C.T., K.H.A. and M.W. contributed to interpretation of the data and drafting of the manuscript.

## Funding

The study has received funding from The Research Foundation of Region Västra Götaland (VGFOUREG‐388361; 474381; 568841), TUA Reasearch Funding (Region Västra Götaland) (TUAGBG‐210211; 364881; 63655; 978440) and the Public Dental Service, Västra Götaland, Sweden.

## Ethics Statement

The field study protocol was approved by the Regional Ethical Review Board, Gothenburg, Sweden (Dnr: 288‐13) and registered at ClinicalTrials.gov (NCT02168621). Written informed consent was obtained from all participants.

## Conflicts of Interest

The authors declare no conflicts of interest.

## Supporting information


**Table S1:** Treatment‐ and travel‐related variables included in the cost analysis. Presented as mean (range). Total number of participants *n* = 615.

**Table S2:** Way of transport mode for CNST and GPIC, presented in percentage (%). Standard costs are by transport mode per kilometre (km), based on national calculations regarding cost for different transport modes†. Also presented is the average cost of transport for both groups.

**Table S3:** The hourly rate for dental hygienists of all Public Dental Service Regions and the average of hourly rate in total, as mean (range).

**Table S4:** Subgroup analysis regarding treatment costs in relation to disease severity at baseline. Mean and 95% CI (*n* = 615).

**Table S5:** Price and quantity table, for subgroup analysis of employees vs. retirees, students and others, consisting of cost items, quantities and values regarding the productivity cost of non‐surgical periodontal treatments (steps 1 to 3, Sanz et al. 2020) (*n* = 615).

## Data Availability

The data that support the findings of this study are available from the corresponding author upon reasonable request. Supporting Information is available on‐line for this article. It includes additional data, methodological details and supporting analyses.

## Endnotes

^1^
Overhead refers to the indirect costs included in the hourly rate, such as administrative services, IT systems, facility operations, and other organisational expenses that support clinical work.

## References

[jcpe70154-bib-0029] Axelsson, P. , B. Nyström , and J. Lindhe . 2004. “The Long‐Term Effect of a Plaque Control Program on Tooth Mortality, Caries and Periodontal Disease in Adults: Aesults After 30 Years of Maintenance.” Journal of clinical periodontology 31, no. 9: 749–757. 15312097 10.1111/j.1600-051X.2004.00563.x

[jcpe70154-bib-0001] Bishop, C. 2021. “Time to Take Gum Disease Seriously: The Societal and Economic Impact of Periodontitis.” Economist Intelligence Unit 2022: 1–46.

[jcpe70154-bib-0002] Botelho, J. , V. Machado , Y. Leira , L. Proenca , L. Chambrone , and J. J. Mendes . 2021. “Economic Burden of Periodontitis in the United States and Europe ‐ An Updated Estimation.” Journal of Periodontology 93: 373–379. 10.1002/JPER.21-0111. 34053082

[jcpe70154-bib-0003] Drummond, M. F. 2015. Methods for the Economic Evaluation of Health Care Programmes, edited by M. F. Drummond , M. J. Sculpher , K. Claxton , G. L. Stoddart , and G. W. Torrance , Fourth ed. Oxford University Press.

[jcpe70154-bib-0004] Drummond, M. , R. Aguiar‐Ibanez , and J. Nixon . 2006. “Economic evaluation.” Singapore Medical Journal 47, no. 6: 456. 16752012

[jcpe70154-bib-0005] Dumitrescu, A. L. 2016. “Editorial: Periodontal Disease – A Public Health Problem.” Frontiers in Public Health 3, no. 278: 278. 10.3389/fpubh.2015.00278. 26779473 PMC4705816

[jcpe70154-bib-0006] Eberhard, J. , S. Jepsen , P. M. Jervøe‐Storm , I. Needleman , and H. V. Worthington . 2015. “Full‐Mouth Treatment Modalities (Within 24 Hours) for Chronic Periodontitis in Adults.” Cochrane Database of Systematic Reviews 4: CD004622. 10.1002/14651858.CD004622.pub3. PMC8687876 25884249

[jcpe70154-bib-0007] Holm, S. 2013. Declaration of Helsinki, 1–4. International Encyclopedia of Ethics. 10.1002/9781444367072.wbiee230.pub2.

[jcpe70154-bib-0008] Husereau, D. , M. Drummond , F. Augustovski , et al. 2022. “Consolidated Health Economic Evaluation Reporting Standards 2022 (CHEERS 2022) Statement: Updated Reporting Guidance for Health Economic Evaluations.” MDM Policy & Practice 7, no. 1: 23814683211061097. 10.1177/23814683211061097. 35036563 PMC8755935

[jcpe70154-bib-0009] Jevdjevic, M. , and S. Listl . 2025. “Global, Regional, and Country‐Level Economic Impacts of Oral Conditions in 2019.” Journal of Dental Research 104, no. 1: 17–21. 10.1177/00220345241281698. 39535193 PMC11662506

[jcpe70154-bib-0010] Kassebaum, N. , E. Bernabé , M. Dahiya , B. Bhandari , C. Murray , and W. Marcenes . 2014. “Global Burden of Severe Periodontitis in 1990‐2010: A Systematic Review and Meta‐Regression.” Journal of Dental Research 93, no. 11: 1045–1053. 10.1177/0022034514552491. 25261053 PMC4293771

[jcpe70154-bib-0011] Kassebaum, N. J. , A. G. C. Smith , E. Bernabé , et al. 2017. “Global, Regional, and National Prevalence, Incidence, and Disability‐Adjusted Life Years for Oral Conditions for 195 Countries, 1990‐2015: A Systematic Analysis for the Global Burden of Diseases, Injuries, and Risk Factors.” Journal of Dental Research 96, no. 4: 380–387. 10.1177/0022034517693566. 28792274 PMC5912207

[jcpe70154-bib-0012] Koshy, G. , Y. Kawashima , M. Kiji , et al. 2005. “Effects of Single‐Visit Full‐Mouth Ultrasonic Debridement Versus Quadrant‐Wise Ultrasonic Debridement.” Journal of Clinical Periodontology 32, no. 7: 734–743. 10.1111/j.1600-051X.2005.00775.x. 15966880

[jcpe70154-bib-0013] Liss, A. , J. L. Wennström , M. Welander , C. Tomasi , M. Petzold , and K. H. Abrahamsson . 2021. “Patient‐Reported Experiences and Outcomes Following Two Different Approaches for Non‐Surgical Periodontal Treatment: A Randomized Field Study.” BMC Oral Health 21, no. 1: 1–13. 10.1186/s12903-021-02001-4. 34911530 PMC8672495

[jcpe70154-bib-0014] Loggner Graff, I. , B. Asklöw , and H. Thorstensson . 2008. “Full‐Mouth Versus Quadrant‐Wise Scaling – Clinical Outcome, Efficiency and Treatment Discomfort.” Swedish Dental Journal 33, no. 3: 105–113. 19994560

[jcpe70154-bib-0015] Passarelli, P. C. , S. Pagnoni , G. B. Piccirillo , et al. 2020. “Reasons for Tooth Extractions and Related Risk Factors in Adult Patients: A Cohort Study.” International Journal of Environmental Research and Public Health 17, no. 7: 2575. 10.3390/ijerph17072575. 32283707 PMC7178127

[jcpe70154-bib-0016] Pattamatta, M. , I. Chapple , and S. Listl . 2025. “The Value‐For Money of Preventing and Managing Periodontitis: Opportunities and Challenges.” Periodontology 2000 98, no. 1: 56–64. 10.1111/prd.12569. 38745388 PMC12842866

[jcpe70154-bib-0017] Righolt, A. , M. Jevdjevic , W. Marcenes , and S. Listl . 2018. “Global‐, Regional‐, and Country‐Level Economic Impacts of Dental Diseases in 2015.” Journal of Dental Research 97, no. 5: 501–507. 10.1177/002203451775057. 29342371

[jcpe70154-bib-0018] Sanz, M. , D. Herrera , M. Kebschull , et al. 2020. “Treatment of Stage I‐III Periodontitis‐The EFP S3 Level Clinical Practice Guideline.” Journal of Clinical Periodontology 47, no. Suppl 22: 4–60. 10.1111/jcpe.13290. 32383274 PMC7891343

[jcpe70154-bib-0019] SBU . 2004. Kronisk Parodontit ‐ Prevention, Diagnostik och Behandling, Statens beredning för medicinsk och social utvärdering. Swedish Council on Health Technology Assessment (SBU).

[jcpe70154-bib-0020] SCB . 2026. “Medellöner i Sverige.” https://www.scb.se/hitta‐statistik/sverige‐i‐siffror/utbildning‐jobb‐och‐pengar/medelloner‐i‐sverige/#Lon_Sverige.

[jcpe70154-bib-0021] SKaPa . 2024. SKaPa Årsrapport 2023: Svenskt kvalitetsregister för Karies och Parodontit (in Swedish). http://www.skapareg.se/other‐language/.

[jcpe70154-bib-0022] Socialstyrelsen . 2011. Nationella riktlinjer för vuxentandvård 2011‐stöd för styrning och ledning. National Board of Health and Wellfare (Socialstyrelsen).

[jcpe70154-bib-0023] Socialstyrelsen . 2022. “National Guidelines for Dental Care (in Swedish).” https://www.socialstyrelsen.se/globalassets/sharepoint‐dokument/artikelkatalog/nationella‐riktlinjer/2022‐9‐8114.pdf (in Swedish) or https://www.socialstyrelsen.se/globalassets/sharepoint‐dokument/artikelkatalog/nationella‐riktlinjer/2022‐9‐8131.pdf (summary in English).

[jcpe70154-bib-0024] Suvan, J. , Y. Leira , F. M. Moreno Sancho , F. Graziani , J. Derks , and C. Tomasi . 2020. “Subgingival Instrumentation for Treatment of Periodontitis. A Systematic Review.” Journal of Clinical Periodontology 47, no. S22: 155–175. 10.1111/jcpe.13245. 31889320

[jcpe70154-bib-0025] The Swedish Public Dental Service . (2025). https://www.folktandvardenstockholm.se/folktandvarden‐sverige/.

[jcpe70154-bib-0026] Tomasi, C. , A. Liss , M. Welander , A. Y. Alian , K. H. Abrahamsson , and J. L. Wennström . 2022. “A Randomized Multi‐Centre Study on the Effectiveness of Non‐Surgical Periodontal Therapy in General Practice.” Journal of Clinical Periodontology 49, no. 11: 1092–1105. 10.1111/jcpe.13703. 35833528 PMC9796759

[jcpe70154-bib-0027] Tonetti, M. S. , S. Jepsen , L. Jin , and J. Otomo‐Corgel . 2017. “Impact of the Global Burden of Periodontal Diseases on Health, Nutrition and Wellbeing of Mankind: A Call for Global Action.” Journal of Clinical Periodontology 44, no. 5: 456–462. 10.1111/jcpe.12732. 28419559

[jcpe70154-bib-0028] Wennström, J. L. , C. Tomasi , A. Bertelle , and E. Dellasega . 2005. “Full‐Mouth Ultrasonic Debridement Versus Quadrant Scaling and Root Planing as an Initial Approach in the Treatment of Chronic Periodontitis.” Journal of Clinical Periodontology 32, no. 8: 851–859. 10.1111/j.1600-051X.2005.00776.x. 15998268

