# Supplementary material for: Cost Consequence Analysis of Non‐Surgical Treatment Approaches for Patients With Periodontitis
Source: J Clin Periodontol. 2026 Jun 19;53(10):1557–64. doi: 10.1111/jcpe.70154 (PMC13581471; doi:10.1111/jcpe.70154)
Supplement: Supplementary file 1 [file JCPE-53-1557-s001.docx]

| Table S2. Way of transport mode for CNST and GPIC, presented in percentage, %. Standard costs by transport mode per kilometer (km), based on national calculations regarding cost for different transport mode†. As well as, presenting the average cost of transports for both groups. | | |
| --- | --- | --- |
| Way of transport, % | CNST | GPIC |
| Car | 76.9 | 72.8 |
| Bus/tram/train | 5.2 | 9.9 |
| Bicycle | 5.2 | 5.5 |
| Walking | 12.7 | 11.8 |
|  |  |  |
| Standard cost by transport mode per kilometer† |  | |
| Car | €0.5/ SEK5.0 | |
| Bus/Tram/Train | €0.1/ SEK1.1 | |
| Walking | €0/ SEK0 | |
| Bicycle | **€0.1/ SEK0.8** | |
|  |  | |
| Average cost of transport for both groups,  mean (range) | €0.4 (0-0.5)/ SEK3.9 (0-5) | |
|  |  | |
| € 1 = SEK 10.84 (in Mars 2025). Abbreviations: €, Euro; SEK, Swedish crowns  †Cost for different transport modes (in Swedish):  <https://bransch.trafikverket.se/contentassets/712f15d62a824319b1815365ef5750dd/berakningshandledning-cykel.pdf>  <https://msverige.se/allt-om-bilen/vad-kostar-det-att-ha-bil/bilkostnadskalkyl/>  <https://autocosts.info/se>  <https://www.trafa.se/etiketter/transportovergripande/transportsektorns-samhallsekonomiska-kostnader-for-2021-13012/>  <https://www.diva-portal.org/smash/get/diva2:1902333/FULLTEXT01.pdf> | | |
