# Supplementary material for: Cost Consequence Analysis of Non‐Surgical Treatment Approaches for Patients With Periodontitis
Source: J Clin Periodontol. 2026 Jun 19;53(10):1557–64. doi: 10.1111/jcpe.70154 (PMC13581471; doi:10.1111/jcpe.70154)
Supplement: Supplementary file 2 [file JCPE-53-1557-s002.docx]

| Table S5. Price and Quantity-table, for subgroup analysis of employees vs. retirees, students and others, consisting of cost items, quantities and values regarding the productivity cost of non-surgical periodontal treatments (step 1 to 3, Sanz et al., 2020) (n=615) | | | | | | | | | | |
| --- | --- | --- | --- | --- | --- | --- | --- | --- | --- | --- |
| **Variables** |  | | **Treatment CNST** | | | **Treatment GPIC** | | | **Incremental (CNST-GPIC)** | |
|  | **Price (P)**  Mean (range) | | **Quantity (Q)** | **Total**  Mean (95% CI) | | **Quantity (Q)** | **Total**  Mean (95% CI) | |  | |
| **Cost item** | (€) | (SEK) |  | (€) | (SEK) |  | (€) | (SEK) | (€) | (SEK) |
|  |  |  |  |  |  |  |  |  |  |  |
| **Productivity cost†**  (n=239 resp 206) | 32.6 | 353.0 | 4.6 h | 150.4 (146.5-154.6) | 1630.7 (1588.3–1676.1) | 4.2 h | 135.7 (133.0-138.5) | 1471.1 (1441.3–1501.0) | +14.7  (9.8–19.7) | +159.6  (106.1–213.2) |
| **Productivity cost‡**  (n= 96 resp 74) | **14.0** | 151.3 | 4.4 h | **62.0 (59.3–64.9)** | 671.9 (643.1–703.4) | 4.0 | **55.7 (53.7–57.7)** | 604.2 (582.5–625.9) | **+6.3**  **(2.8–9.7)** | **+67.7**  **(30.8–104.7)** |
| **Total** |  |  |  | 212.4 | 2302.6 |  | 191.4 | 2075.3 | +21.0 | +227.3 |
| GPIC, guided periodontal infection control; CNST, conventional non-surgical therapy.  € 1 =SEK 10.84 (in Mars 2025). Abbreviations: €, Euro; SEK, Swedish crowns  †Individuals in paid employment  ‡ Retired, students and others | | | | | | | | | | |
