# Supplementary material for: Cost Consequence Analysis of Non‐Surgical Treatment Approaches for Patients With Periodontitis
Source: J Clin Periodontol. 2026 Jun 19;53(10):1557–64. doi: 10.1111/jcpe.70154 (PMC13581471; doi:10.1111/jcpe.70154)
Supplement: Supplementary file 3 [file JCPE-53-1557-s003.docx]

Table S3. The hourly rate for Dental hygienists of all Public Dental Service Regions and the average of hourly rate in total; mean (range).

| **Public Dental Service, regions in Sweden** | **Hourly rate (€)** | **Hourly rate (SEK)** |
| --- | --- | --- |
| Blekinge | 144 | 1573 |
| Dalarna | 220 | 2400 |
| Gotland | 125 | 1366 |
| Gävleborg | 134 | 1456 |
| Halland | 122 | 1325 |
| Jämtland | - | - |
| Jönköping | 137 | 1495 |
| Kalmar | 142 | 1547 |
| Kronoberg | 119 | 1300 |
| Norrbotten | 122 | 1325 |
| Skåne | 114 | 1240 |
| Stockholm | 125 | 1360 |
| Sörmland | 153 | 1670 |
| Uppsala | 119 | 1300 |
| Värmland | 126 | 1373 |
| Västerbotten | 125 | 1360 |
| Västernorrland | 130 | 1417 |
| Västmanland | 123 | 1339 |
| Västra Götaland | 135 | 1465 |
| Örebro | 126 | 1375 |
| Östergötland | 127 | 1385 |
| **Mean (range)** | **134** (114–220) | 1454 (1240-2400) |
| € 1= SEK 10.84 (in Mars 2025). Abbreviations: €, Euro; SEK, Swedish crowns  The hourly rate reflects the full cost of providing one hour of clinical work, including staff-related expenses, clinic infrastructure, and organizational overhead. Overhead refers to the indirect costs included in the hourly rate, such as administrative services, IT systems, facility operations, and other organizational expenses that support clinical work. | | |
