# Supplementary material for: Cost Consequence Analysis of Non‐Surgical Treatment Approaches for Patients With Periodontitis
Source: J Clin Periodontol. 2026 Jun 19;53(10):1557–64. doi: 10.1111/jcpe.70154 (PMC13581471; doi:10.1111/jcpe.70154)
Supplement: Supplementary file 4 [file JCPE-53-1557-s004.docx]

| Table S1. Treatment and travel related variables included in the cost analysis. Presented in mean (range). Total number of participants n=615. | | |
| --- | --- | --- |
| Variables | CNST (n=335) | GPIC (n=280) |
| Total treatment time^†^, in minutes | 160.9 (52.0-490.0) | 134.0 (20.5-250.0) |
| Total visits at the dental office in the initial phase^§^, number of visits | 4.1 (1-9) | 4.0 (1-6) |
| Traveled distance to dental office for treatment during the study, in kilometers | 68.0 (16.6–149.4) | 65.5 (16.6–99.6) |
|  |  |  |
|  | CNST  (n=239) | GPIC  (n=206) |
| Total treatment time^†^, in minutes employed | 163.0 (52.0-438.0) | 135.9 (20.50-250.0) |
| Total travel time, in minutes (in paid employment) | 114.2 (0–600.0) | |
| Total treatment and travel time, in minutes (in paid employment) | 277.2 (166.2-604.2) | 250.04 (134.7-364.2) |
|  | (n= 96) | (n= 74) |
| Total treatment time^†^, in minutes retired | 155.5 (60.0-360.0) | 128.6 (45.0-240.0) |
| Total travel time, in minutes (retired, students and others) | 110.95 (0–480.0) | |
| Total treatment and travel time, in minutes (retired, students and others) | 266.4 (171.0-471.0) | 239.6 (156.0-351.0) |
| CNST, conventional non-surgical therapy; GPIC, guided periodontal infection control.  †Total treatment time included patient education (information, instruction and motivation) as well as mechanical instrumentation of affected teeth and root surfaces. | | |
