# Supplementary material for: Cost Consequence Analysis of Non‐Surgical Treatment Approaches for Patients With Periodontitis
Source: J Clin Periodontol. 2026 Jun 19;53(10):1557–64. doi: 10.1111/jcpe.70154 (PMC13581471; doi:10.1111/jcpe.70154)
Supplement: Supplementary file 5 [file JCPE-53-1557-s005.docx]

| Table S4. Subgroup analysis regarding treatment costs in relation to disease severity at baseline. Mean, %95CI. (n=615) | | | | | | |
| --- | --- | --- | --- | --- | --- | --- |
| **Periodontal disease severity**  (at baseline) | **CNST** | | **GPIC** | | **Incremental (CNST-GPIC)** | |
|  | (€) | (SEK) | (€) | (SEK) | (€) | (SEK) |
| **Stage II** (PPD≤5 mm) | 335.8 (297.6-376.2) | 3639.7 (3225.8–4078.1) | 298.2 (275.6-321.0) | 3232.4 (2987.9–3479.2) | +37.6  (-5.5 to 80.7) | +407.3  (-59.9 to 874.5) |
| **Stage III-IV** (≤3 teeth with PPD ≥6 mm) | 335.7 (312.0-360.7) | 3639.1 (3392.7–3910.1) | 293.4 (276.6-310.5) | 3180.3 (2998.3–3366.3) | +42.3  (13.5 – 71.2) | +458.8  (146.5 – 771.0) |
| **Stage III-IV** (≥4 teeth with PPD ≥6 mm) | 376.4 (358.5-395.6) | 4080.3 (3886.3–4288.0) | 304.7 (289.1-320.0) | 3302.5 (3133.8–3468.5) | +71.8  (47.5 – 96.0) | +777.8  (514.7 -1040.9) |
| CNST, conventional non-surgical therapy; GPIC, guided periodontal infection control.  € 1= SEK 10.84 (in Mars 2025).  Abbreviations: BoP, bleeding on probing; €, Euro; SEK, Swedish crowns | | | | | | |
